# Dihydrophenanthrenes from a Sicilian Accession of *Himantoglossum robertianum* (Loisel.) P. Delforge Showed Antioxidant, Antimicrobial, and Antiproliferative Activities

**DOI:** 10.3390/plants10122776

**Published:** 2021-12-15

**Authors:** Natale Badalamenti, Sabino Russi, Maurizio Bruno, Viviana Maresca, Alessandro Vaglica, Vincenzo Ilardi, Anna Zanfardino, Michela Di Napoli, Mario Varcamonti, Piergiorgio Cianciullo, Giovanni Calice, Simona Laurino, Geppino Falco, Adriana Basile

**Affiliations:** 1 Department of Biological, Chemical and Pharmaceutical Sciences and Technologies (STEBICEF), Università degli Studi di Palermo, Viale delle Scienze, ed. 17, 90128 Palermo, Italy; maurizio.bruno@unipa.it (M.B.); alex.vaglica@gmail.com (A.V.); vincenzo.ilardi@unipa.it (V.I.); 2 IRCCS CROB—Referral Cancer Center of Basilicata, 85028 Rionero in Vulture, Italy; sabino.russi@crob.it (S.R.); giovanni.calice@crob.it (G.C.); 3 Centro Interdipartimentale di Ricerca “Riutilizzo bio-based degli scarti da matrici agroalimentari” (RIVIVE), Università di Palermo, 90128 Palermo, Italy; 4 Department of Biology, University of Naples Federico II, 80126 Naples, Italy; anna.zanfardino@unina.it (A.Z.); michela.dinapoli@unina.it (M.D.N.); mario.varcamonti@unina.it (M.V.); piergiorgio.cianciullo@unina.it (P.C.); geppino.falco@unina.it (G.F.); adbasile@unina.it (A.B.)

**Keywords:** dihydrophenanthrenes, *Himantoglossum robertianum*, NMR, antioxidant enzymes, antimicrobial activity, anti-proliferative activity, proapoptotic activity

## Abstract

The peculiar aspect that emerges from the study of Orchidaceae is the presence of various molecules, which are particularly interesting for pharmaceutical chemistry due to their wide range of biological resources. The aim of our study was to investigate the properties of two dihydrophenanthrenes, isolated, for the first time, from *Himantoglossum robertianum* (Loisel.) P. Delforge (Orchidaceae) bulbs and roots. Chemical and spectroscopic study of the bulbs and roots of *Himantoglossum* *robertianum* (Loisel.) P. Delforge resulted in the isolation of two known dihydrophenanthrenes—loroglossol and hircinol—never isolated from this plant species. The structures were evaluated based on ^1^H-NMR, ^13^C-NMR, and two-dimensional spectra, and by comparison with the literature. These two molecules have been tested for their possible antioxidant, antimicrobial, antiproliferative, and proapoptotic activities. In particular, it has been shown that these molecules cause an increase in the activity of superoxide dismutase (SOD), catalase (CAT), and glutathione S-transferase (GST) in polymorphonuclear leukocytes (PMN); show antimicrobial activity against *Escherichia coli* and *Staphylococcus aureus*, and have anti-proliferative effects on gastric cancer cell lines, inducing apoptosis effects. Therefore, these two molecules could be considered promising candidates for pharmaceutical and nutraceutical preparations.

## 1. Introduction

Orchids, the representatives of the Orchidaceae family, are distributed across all continents, except in Antarctica, and they are more prevalent in tropical and subtropical regions. The Orchidaceae family, widely used for ornamental purposes, assembles species with a wide range of applications, such as cosmetics, perfumes, and pharmaceuticals [1].

The genus *Himantoglossum* Spreng, belonging to the Orchidaceae family, was divided, based on molecular and morphometric investigations, into three subgenera, *Himantoglossum*, *Barlia,* and *Comperia*. subgen. *Barlia* (Parl.) enumerates only two species, *Himantoglossum metlesicsianum* (W.P. Teschner) P. Delforge [2], endemic to the Canary Islands and *H. robertianum* (Loisel.) P. Delforge [2] (Syn. *Aceras longibracteatum* Rchb.f.; *Barlia longibracteata* (Rchb.f.) Parl.; *Barlia robertiana* (Loisel.) Greuter; *Himantoglossum longibracteatum* (Rchb.f.) Schltr.; *Loroglossum longibracteatum* (Rchb.f.) Moris ex Ardoino; *Orchis robertiana* Loisel.) [3], with Steno-Mediterranean distribution, which extends from Portugal to Anatolia [4].

*H. robertianum* is a large orchid, whose flowering occurs in Sicily during the winter months (December-February). It presents with 2(–3) large ovoid RhizoTubes from which numerous and thick roots branch off, emitting a robust stem that often exceeds up to 10 mm in diameter. The basal leaves are large, up to 30 cm in length and 10 cm in width, and glossy. The plant is able to grow even up to 1700 m of altitude and is called “giant orchid”, as it exceeds most of the European wild orchids in height [5]. The inflorescence (up to 65 flowers), more or less cylindrical, is dense and rich. The color of the inflorescence varies from greenish–white to purplish red [4]. In Sicily, the species is frequently found along roadsides, uncultivated fields, garrigue, and scrubs, particularly on the chalky substrates of central-southern Sicily, up to 1000 m s.l.m.

The species, in Italy, is protected at the national level but, in some areas around Catania (Sicily), the plant is roasted and eaten [6]. In Turkey, there are several cases of cultivation for ornamental purposes [7], while in Iran, food uses are reported for the species *H. hircinum* (L.) Spreng. (Syn. *H. affine*), *H. jankae* Somlyay, Kreutz and Óvári and *H. comperianum* (Steven) P. Delforge, which they are used to produce salep flour, drinks, and ice cream [8].

The peculiar aspect that emerges from the study of Orchidaceae is the presence of various derivatives based on stilbenes [9], particularly interesting for pharmaceutical chemistry due to their wide range of biological resources [10]. The molecules frequently found in this family are stilbenes, bibenzyls, or dihydrostylbenes, bis(bibenzyls), phenanthrenes, and 9,10-dihydrophenanthrenes. Phenanthrenes, secondary metabolites of many higher plants, are widely conjugated aromatic compounds, and they essentially differ in the position of the different substituents in the rings and in the presence of ketone and hydroxyl groups. A large proportion of these metabolites were found in *Bletilla, Bulbophyllum, Coelogyna, Cymbidium, Dendrobium, Ephemerantha, Epidendrum, Eria,* and *Maxillaria* genera, all belonging to Orchidaceae family [10]. Many natural phenanthrenes also exist as 9,10-dihydro-, or dehydro derivatives. Many reports have described the isolation and structure characterization of a significant number of phenanthropyrans and stilbenoids in different orchids [11,12,13].

The literature emphasized excellent properties of phenanthrene derivatives, exhibiting diverse and promising biological activities, including anti-inflammatory, antimicrobial, spasmolytic, anti-platelet, antioxidant, and anti-allergic activities [14]. Moreover, the cytotoxic effects of phenanthrene derivatives against the growth of several human cancer cell lines were reported [15].

The literature is lacking in the study of the phytochemical and biological properties of *H. robertianum* [16]. The only one study to perform phytochemical analyses on hydroalcoholic extract obtained from *H. robertianum* flowers, highlighted how flavones and flavan-3-oils had represented the most abundant compounds (42.91%), followed by scopoletin (33.79%) and phenolic acids (23.3%) [16]. The extract also showed excellent antioxidant power, classifying oxygen radical absorbance capacity (ORAC) < ferric-reducing antioxidant power (FRAP) < trolox equivalent antioxidant capacity (TEAC) < whitening of -carotene < 2,2-diphenyl-1-picrylhydrazyl (DPPH) < chelation of iron.

Consequently, in the frame of our ongoing research on endemic Sicilian plants [17,18,19] and on the biological activity [20,21,22], the aim of our study was to investigate the biological properties of two dihydrophenanthrenes, loroglossol and hircinol, isolated, for the first time, from *Himantoglossum robertianum* (Loisel.) P. Delforge bulbs and roots, testing the activity of superoxide dismutase (SOD), catalase (CAT), and glutathione S-transferase (GST) enzymes in polymorphonuclear leukocytes (PMN); antimicrobial activity against *Escherichia coli* DH5α and *Staphylococcus aureus* ATCC 6538P, antiproliferative and proapoptotic activity on gastric cancer cell lines.

## 2. Results and Discussion

### 2.1. Chemical Profiling

**Figure 1:**
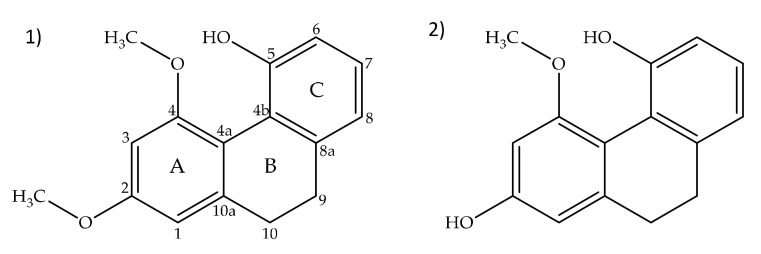
Structures of loroglossol (**1**) and hircinol (**2**).

The CHCl_3_ extract of the air-dried bulbs and roots from *H. robertianum* was subjected to several chromatographic separations to give two compounds: loroglossol (**1**) and hircinol (**2**) (Figure 1), they were identified by ^1^H-NMR, ^13^C-NMR, 2D-NMR, and HPLC-MS.

**Figure 2:**
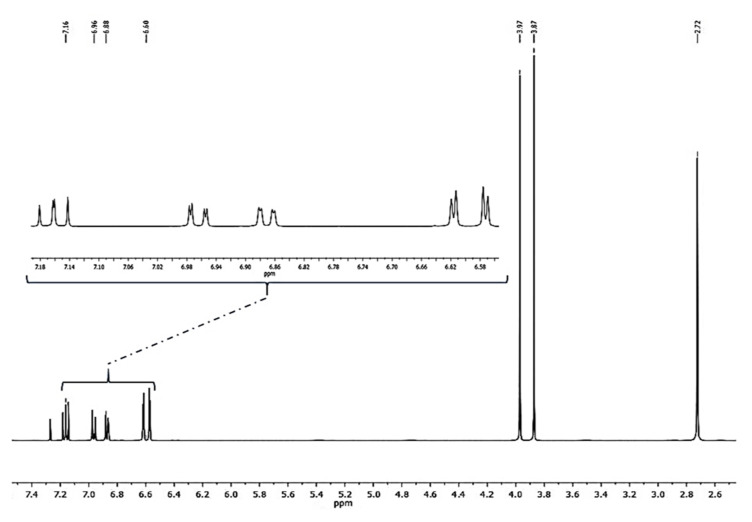
^1^H-NMR spectrum (400 MHz, CDCl_3_) of loroglossol.

**Figure 3:**
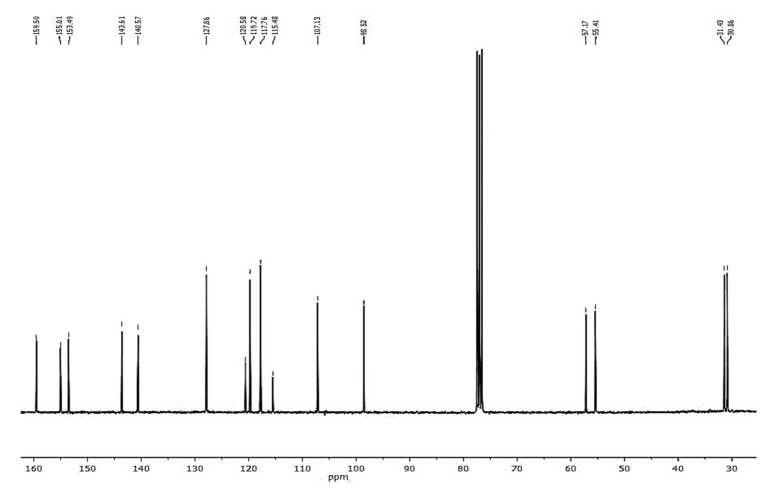
^13^C-NMR spectrum (100 MHz, CDCl_3_) of loroglossol.

**Figure 4:**
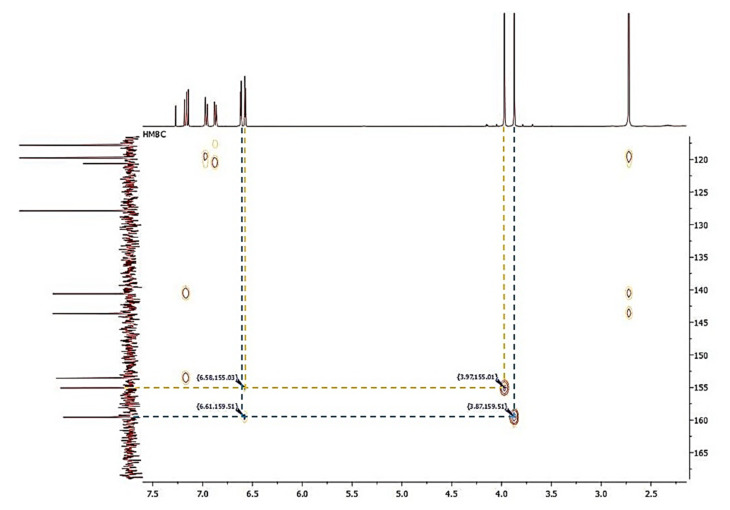
HMBC spectrum showing correlations in loroglossol.

Compound **1** was obtained as a colorless amorphous powder. HPLC-MS showed a molecular ion at *m*/*z* 257.1181 [M + H]^+^ (calcd. for C_16_H_16_O_3_+[H]^+^, *m*/*z* 257.1172). The ^1^H-NMR spectrum of **1** (Figure 2) showed characteristic signals for a 9,10-dihydrophenanthrene: 4 aliphatic protons H-9 and H-10 (*δ* = 2.72 ppm, br s, 4H), which confirm the absence of double bond between C-9 and C-10; the coupling between H-1 and H-3 protons (dd, *J* = 2.5 Hz) for a meta-di-substituted aromatic ring, highlighted the presence of two methoxy-substituents on C-2 and C-4 (*δ*C = 159.5 and 155.0 ppm, respectively) (Figure 3). The HMBC correlations of H-1 (*δ* = 6.62 ppm) with the carbons C-2 (*δ* = 159.5 ppm) and the correlation of C-2 with methoxy protons -OC*H_3_* at 3.97 ppm established the methoxy group on C-2 carbon (Figure 4). The presence of other substituents (a methoxy group on C-4) was confirmed by correlations between H-3 and C-4, and C-4 with methoxy protons at 3.87 ppm (Figure 4). The aromatic signals between 6.88 and 7.16 ppm showed a mono-substituted (hydroxy group) on C-5. The values of chemical shift (*δ*C) presented in literature were registered only in CD_3_OD [23], but our correct structural evaluation of **1** has been confirmed by 2D-NMR and HPLC-MS spectra.

**Figure 5:**
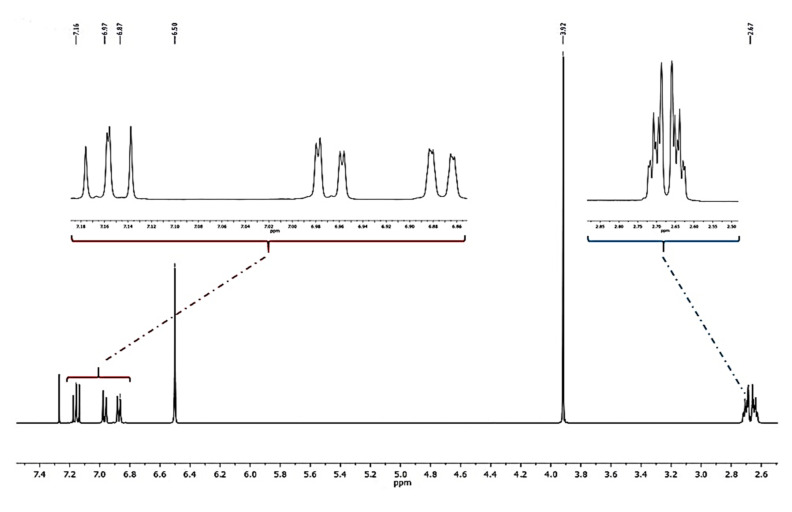
The 1H-NMR spectrum (400 MHz, CDCl3) of hircinol.

**Figure 6:**
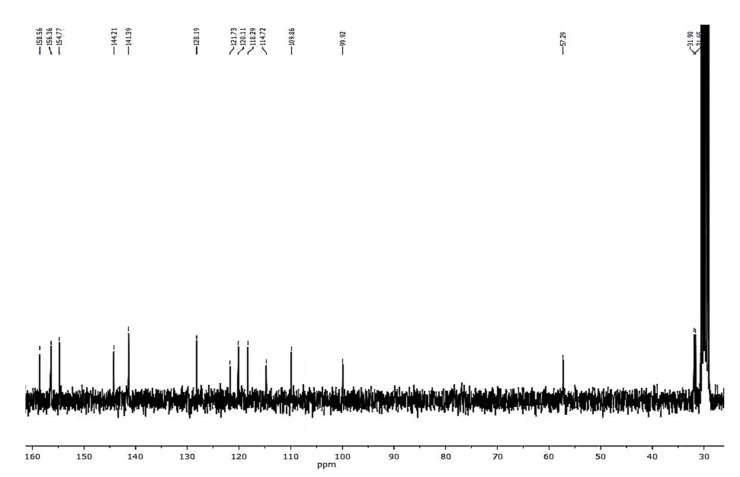
^13^C-NMR spectrum (100 MHz, (CD_3_)_2_CO) of hircinol.

HPLC-MS of amorphous powder compound **2** showed a molecular ion at *m*/*z* 243.0948 [M + H]^+^ (calcd. for [M + H]^+^, *m*/*z* 243.1016), which agreed with the molecular formula C_15_H_14_O_3_. It is a saturated phenanthrene in position C9-C10 (31.7 and 31.9 ppm, respectively) with two hydroxyl groups in position C-2 (158.6 ppm) and C-5 (154.8 ppm) and a single methoxy group in position C-4 (156.4 ppm). The absence of double bond was confirmed by a resonance system at 2.67 ppm (4H, m, H_2_-9,-10), while the presence of a single methoxy-group was verified from signal at 3.92 ppm (3H, s, -OC*H_3_*). The C ring showed the same proton signals of compound **1** (Figure 5 and Figure 6). The impossibility of exploiting the HMBC correlations (due to the overlapping between the H-1 and H-3 protons) did not allow the exact identification of the molecule, which was verified by exact comparison with the literature [24].

Loroglossol and hircinol were first isolated in 1963 from *Loroglossum hircinum* (L.) Rich (Orchidaceae) [25,26], although their structures were definitely elucidated several years later by Fish et al. [27] and Letcher et al. [28]. Later, loroglossol was isolated from *Orchis papilionacea* L. (Orchidaceae) [29] and together with hircinol in *Sobralia violocea* Linden (Orchidaceae) [30]. On the other hand, hircinol has been detected in many other taxa, such as *Flickingeria fimbriata* (Bl.) Hawkes [31], *Pholidota articulata* Lindl. [32], *Pholidota chinensis* Lindl. [33], *Liparis japonica* (Miq.) Maxim [34], *Liparis regnieri* Finet [35,36], *Anoectochilus roxburghii* (Wall.) Lindl. [37], *Pleione bulbocodioides* (Franch.) Rolfe [9], several species of *Dendrobium* [38,39,40,41,42,43,44,45], all belonging to Orchidaceae, and in *Dioscorea rotundata* Poir [24] and *Dioscorea opposita* Thunb. [46] of the Dioscoreaceae family.

### 2.2. Antioxidant Enzymes Activity Measure in PMN Cells

We evaluated the antioxidant activity of loroglossol and hircinol by measuring the activity of superoxide dismutase (SOD), catalase (CAT), and glutathione S-transferase (GST) enzymes in polymorphonuclear leukocytes (PMNs) treated with these two molecules.

**Figure 7:**
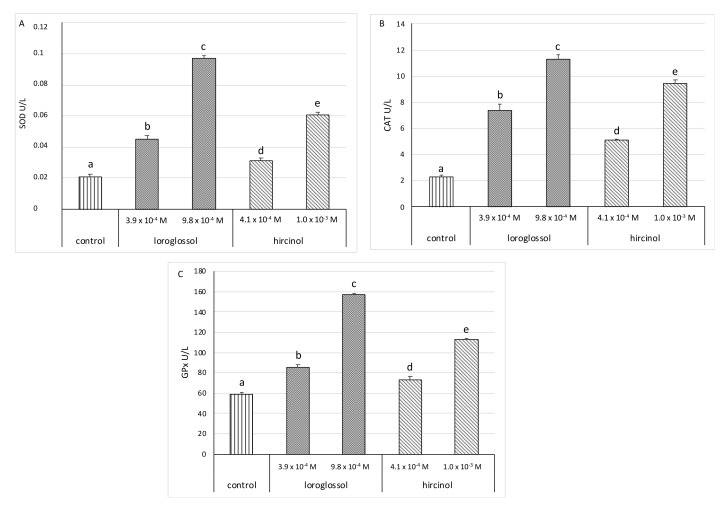
Effect of the loroglossol and hircinol on activities of antioxidant enzymes in polymorphonuclear cells. (**A**) Superoxide dismutase; (**B**) catalase; (**C**) glutathione peroxidase. Data were presented as mean and standard error and they were analyzed with a paired *t*-test. Bars not accompanied by the same letter were significantly different at *p* < 0.05.

The activity of antioxidant enzymes in PMN cells increased after treatment with both loroglossol and hircinol compared to the control (untreated samples) (Figure 7). In particular, the activity of the enzymes SOD, CAT, and GPX increases with increasing concentration. Furthermore, the PMN cells treated with loroglossol have a higher enzymatic activity than the PMN cells treated with hircinol.

Our results demonstrate, for the first time, the ability of these two molecules to increase the activity of antioxidant enzymes in PMNs. Furthermore, these results are consistent with those obtained by Shuang-shuang et al. [15], which demonstrated the excellent properties of phenanthrene derivatives exhibiting diverse and promising biological activities, including antioxidant activities. Moreover, the authors of [47] show how compounds extracted from *Dendrobium pachyglossum* (Orchidaceae), such as dendropachol, cause an increase in CAT and GST n in H_2_O_2_-Treated HaCaT Keratinocytes. This work is also consistent with the study by [48], showing that bibenzyl-dihydrophenanthrene from D. parishii can improve the activities of antioxidant enzymes (GPx and CAT).

However, the antioxidant properties of essential oils cannot be assessed by simply looking at the increased activity of antioxidant enzymes. On the other hand, the increase in their activity is linked to oxidative stress. Generally, the activity of the enzymes CAT, SOD, and GST increases following an increase in ROS production, in order to counteract the negative effects induced by stress, as reported in [49]. In this case, these are not stressful conditions, but we suggest that an increase in these enzymes may indicate an increase in their antioxidant properties.

### 2.3. Antimicrobial Assays

In order to evaluate the antibacterial activity, first, an antimicrobial test (Kirby–Bauer assay) was performed with both molecules on two different bacterial strains, a Gram-negative *E. coli* DH5α and a Gram-positive *S. aureus* ATCC 6538P. *E. coli* represents a model strain to test antimicrobial activity, while *S. aureus* is an important human pathogen that is responsible for most of the bacterial skin and soft tissue infections in humans too. This strain can also become more invasive and cause life-threatening infections, such as bacteremia, pneumonia, abscesses of various organs, meningitis, osteomyelitis, endocarditis, and sepsis. These infections represent a major public health threat because of their considerable number and spread [50]. Both molecules exhibited an inhibition halo against the strains, almost comparable to the antibiotic control (Supplementary Materials Figure S1). We used ampicillin as a positive control, in order to inhibit *E. coli* and *S. aureus* cell growth. No growth inhibition was seen with the DMSO used to dilute oils.

**Figure 8:**
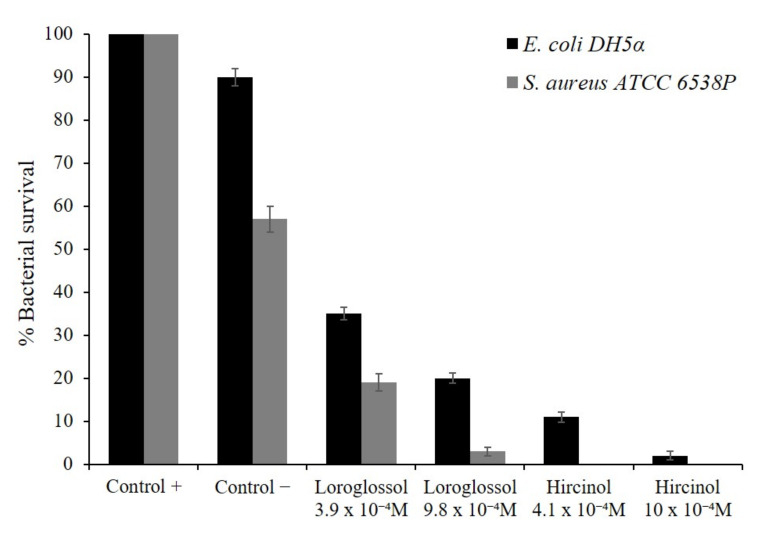
Antibacterial activity of loroglossol and hircinol evaluated by colony count assay, against *E. coli* DH5α and *S. aureus* ATCC 6538P at different concentrations. Untreated cells represent positive control while bacterial cells with dimethyl sulfoxide represent the negative one. Each bar is the average of three different experiments (*p* value is <0.05).

These first experiments allowed us to deepen the study on loroglossol and hircinol antimicrobial activity. Using the same indicator strains as in previous experiments, we performed another assay in determining the substance antimicrobial efficiency by bacterial counts. Figure 8 shows that both molecules extracted from orchids possess good antimicrobial activity, more directly towards *S. aureus*. In particular, hircinol is very effective against the Gram-negative and Gram-positive strains, even at relatively low concentrations. Subsequently antimicrobial activity of loroglossol and hircinol was analyzed accordingly to the broth microdilution method. By performing this assay, minimal inhibitory concentration values were found to be comprised between 3.9 × 10^−4^ M and >1.2 × 10^−3^ M against the tested strains (Supplementary Materials Table S1). Other phenanthrene derivatives are reported in the literature. In particular, six biphenanthrenes extracted from *Bletilla striata* (Orchidaceae) show antimicrobial activity (8–128 µg/mL) against different bacterial strains [51]. Instead, two new dihydrophenanthrofurans and two new bisbibenzyl derivatives isolated from *Dendrobium nobile*, were evaluated against Gram-positive bacterial strains *Staphylococcus aureus*, *Bacillus subtilis,* and Gram-negative bacteria, *Pseudomonas aeruginosa*, *Escherichia coli*, by a microdilution technique, but neither was active [52]. Further studies will be needed to determine the mechanism of action of these substances as a potential antimicrobial agent.

### 2.4. Anti-Proliferative Effects on Gastric Cancer Cell Lines

**Figure 9:**
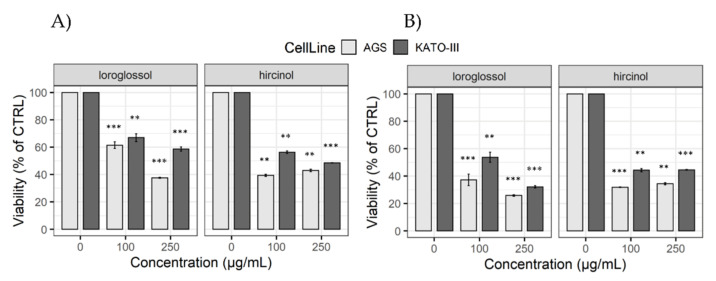
Effect of loroglossol and hircinol on cell proliferation. AGS and KATO-III cells were treated for 24 h (**A**) and for 48 h (**B**). Cell proliferation was determined by MTS assay. Data were expressed as % of vehicle control viability. Data were presented as mean and standard error of three individual experiments and analyzed with one sample-t test. ** *p* < 0.005; *** *p* ≤ 0.0001.

Since the literature reported the cytotoxic activity of extracts from several orchid plants, rich in phenanthrene and dihydrophenanthrene compounds [53,54], we evaluated the effect of loroglossol and hircinol on viability of two different gastric cancer (GC) cell lines (AGS, and KATO-III) through MTS assay. Results were reported as a percentage of control viability (cells exposed to DMSO). Both compounds significantly reduced viability of gastric cancer cell lines. Interestingly, we observed a decrease of about 40% after 24 h of exposure to 100 µg/mL (3.9 × 10^−4^ M for loroglossol: AGS = 61.4 ± 2.4, *p* < 0.0001; KATO-III = 66.9 ± 2.8, *p* = 0.0003 and 4.1 × 10^−4^ M for hircinol: AGS = 39.4 ± 0.8, *p* = 0.0002; KATO-III = 56.3. ± 0.9, *p* = 0.0004) (Figure 9).

Moreover, the effect on cell viability was time-dependent. In particular, long exposure (48 h) to loroglossol at 3.9 × 10^−4^ M showed the maximum anti-proliferative effect, determining an additional viability reduction of about 20% in both cell lines (24 h vs. 48 h, AGS: *p* = 0.002 and KATO-III: *p* = 0.02). Similarly, treatment with hircinol at 4.1 × 10^−4^ M for 48 h decreased cell viability for an additional 10% (24 h vs. 48 h, AGS: *p* = 0.007 and KATO-III: *p* < 0.001). Between the two GC cell lines, AGS was the most sensitive to both compounds, as reported in Table 1.

This evidence could resemble the features of a cell line’s originating tumor, being AGS derived from primary and KATO-III from metastatic GC.

These results match those previously reported by other researchers, which highlighted an anti-proliferative activity of phenanthrenes and 9,10-dihydrophenanthrenes on several cancer cell lines derived from different tumor types, such as leukemia, breast, colon, prostate, and lung cancer [10,55]. Notably, several papers reported that similar molecules, at comparable doses, are effective on cancer cells and well tolerated by normal cells, suggesting a possible selective toxicity [56,57,58,59].

### 2.5. Apoptosis-Inducing Effect on Gastric Cancer Cell Lines

**Figure 10:**
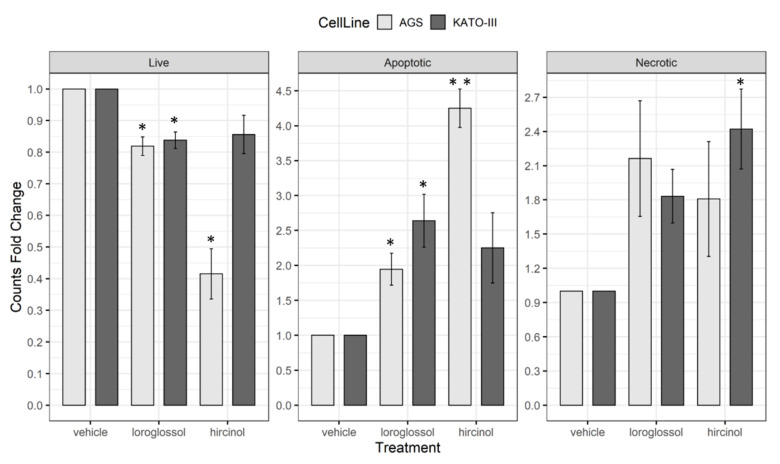
Loroglossol and hircinol induced apoptosis in GC cells. AGS and KATO-III cells were treated with 100 µg/mL (3.9–4.1 × 10^−4^ M) for 48 h. The amount of live, apoptotic and necrotic cells was determined by flow cytometry. The relative counts were estimated in comparison to the vehicle controls. Data were presented as mean and standard error of three individual experiments and were analyzed with one-sample *t*-test. * *p* < 0.05; ** *p* < 0.005.

Further characterization of anti-proliferative effects of loroglossol and hircinol were assessed through flow cytometry analyses; in particular, it was taken in consideration whether treated cells underwent apoptotic cell death. Based on MTS results, apoptosis was evaluated after 48 h of exposure to 100 µg/mL (3.9–4.1 × 10^−4^ M) of both compounds. As showed in Figure 10, a decreased number of live cells was observed in both cell lines loroglossol, AGS: 0.82 ± 0.03, *p* = 0.03 and KATO-III 0.84 ± 0.03, *p* = 0.03; hircinol, AGS: 0.42 ± 0.8, *p* = 0.04 and KATO-III 0.86 ± 0.06, *p* = 0.16).

The assay confirmed the high sensitivity of AGS cells that showed a significant decrease in live cells for both compounds as compared to KATO-III cells. Interestingly, this finding reflects the drug resistant phenotype described in other reports [60,61]

Analyzing the apoptotic cells, it was observed that loroglossol treatment determined a significant increase of apoptosis in both AGS (1.95 ± 0.23, *p* = 0.03) and KATO-III (2.64 ± 0.38, *p* = 0.02) cells, while the treatment with hircinol caused a significant increase of apoptosis only in AGS cell line (4.25 ± 0.28, *p* = 0.002). Necrotic cells were significant increased only in KATO-III cells treated with hircinol. These data showed the apoptotic effects of loroglossol and hircinol on two different gastric cancer cell lines highlighting an anti-tumor potential of these two molecules.

In line with these results, an apoptogenic effect of orchid’s phenanthrene derivatives on various human cancer cells, including osteosarcoma cells [62], breast cancer cells [63], and gastric cancer cells [64], has been reported.

Moreover, several compounds isolated from orchids have demonstrated significant anticancer activities [65]. The isolation of orchid extract whit cytotoxic potential could lay the bases for their chemical engineering into more effective anticancer drugs. This kind of orchid compound, being semi-synthetic, could help to reduce adverse reactions associated with current cancer treatment [66].

## 3. Materials and Methods

### 3.1. Plant Materials

Bulbs and roots of *H. robertianum* were collected from wild populations growing at Sutera (37°30′49′′ N, 13°45′112′′ E, 468 m s.l.m.), in the province of Caltanissetta, Sicily, Italy, in February 2020, and identified by Professor Vincenzo Ilardi (Università degli Studi di Palermo). A voucher specimen was deposited at Department STEBICEF, University of Palermo, Italy, under number (PAL109715).

### 3.2. Extraction and Isolation

The bulbs and roots of *H. robertianum* (315 g), cut in small pieces, were lyophilized, and extracted with CHCl_3_ (3 × 0.5 L). The resulting extracts were evaporated to dryness, to yield 4.5 g of residue separated by silica gel column chromatography with hexane-EtOAc (60:40) eluent to give the main fractions, A–E. Each fraction was rechromatographed by silica gel column chromatography in CHCl_3_-MeOH (98:2) to afford: from fraction A, loroglossol (23 mg); from fraction D, hircinol (28 mg).

### 3.3. Spectroscopic Data

#### 3.3.1. Loroglossol

Colorless needles; ^1^H-NMR (400 MHz, CDCl_3_): *δ* = 2.72 (4H, br s, H_2_-9,-10), 3.87 (3H, s, -OC*H_3_*), 3.97 (3H, s, -OC*H_3_*), 6.57 (1H, d, *J* = 2.5 Hz, H-3), 6.62 (1H, d, *J* = 2.5 Hz, H-1), 6.88 (1H, dd, *J* = 7.2, 1.4 Hz, H-8), 6.96 (1H, dd, *J* = 8.1, 1.4 Hz, H-6), 7.16 (1H, dd, *J* = 8.1, 7.2 Hz, H-7); ^13^C-NMR (100 MHz, CDCl_3_): *δ* = 107.1 (C-1), 159.5 (C-2), 98.5 (C-3), 155.0 (C-4), 153.5 (C-5), 117.8 (C-6), 127.9 (C-7), 119.7 (C-8), 30.9 (C-9), 31.4 (C-10), 115.5 (C-4a), 120.6 (C-4b), 140.6 (C-8a), 143.6 (C-10a), 55.4 (C2-O*C*H_3_), 57.2 (C4-O*C*H_3_); ESIMS *m*/*z* 257.1181 [M + H]^+^, (calcd. for C_16_H_16_O_3_+[H]^+^, *m*/*z* 257.1172).

#### 3.3.2. Hircinol

Amorphous powder; ^1^H-NMR (400 MHz, CDCl_3_): *δ* = 2.67 (4H, m, H_2_-9,-10), 3.92 (3H, s, -OC*H_3_*), 6.50 (2H, br s, H-1, H-3), 6.87 (1H, dd, *J* = 7.3, 1.4 Hz, H-8), 6.97 (1H, dd, *J* = 8.1, 1.4 Hz, H-6), 7.16 (1H, dd, *J* = 8.1, 7.3 Hz, H-7); ^13^C-NMR (100 MHz, [(CD_3_)_2_CO]: *δ* = 109.9 (C-1), 158.6 (C-2), 99.9 (C-3), 156.4 (C-4), 154.8 (C-5), 118.3 (C-6), 128.2 (C-7), 120.1 (C-8), 31.7 (C-9), 31.9 (C-10), 114.7 (C-4a), 121.7 (C-4b), 141.4 (C-8a), 144.2 (C-10a), 57.3 (C2-O*C*H3); ESIMS *m*/*z* 243.0948 [M + H]^+^, (calcd. for C_15_H_14_O_3_+[H]^+^, *m*/*z* 243.1016).

### 3.4. General Experimental Procedures

Column chromatography was performed using silica gel (70-230 mesh ASTM, Merck No. 7734) deactivated with 15% deionized water. Lyophilization was conducted using CoolSafe instrument (4-15L Freeze Dryers). The NMR spectra were recorded on a Bruker Avance II instrument (400 MHz for ^1^H-NMR and 100 MHz for ^13^C-NMR). The thin-layer chromatography used aluminum oxide 60 F254 neutral (Merck KGaA, Darmstadt, Germany). Mass spectrum was obtained using a HPLC/ESI/Q-TOF HRMS apparatus. HPLC conditions were as follows: water, acetonitrile, and formic acid were of HPLC/MS grade; the HPLC system was an Agilent 1260 Infinity; a reversed-phase C18 column (ZORBAX Extended-C_18_ 2.1 × 50 mm, 1.8 μm) with a Phenomenex C18 security guard column (4 × 3 mm) were used; the flow-rate was 0.4 mL/min, and the column temperature was set to 30 °C. The mass spectra was recorded using an Agilent 6540 UHD accurate-mass Q-TOF spectrometer equipped with a Dual AJS ESI source working in both negative and positive modes. All solvents used were of analytical grade (Honeywell, Charlotte, NC, USA).

### 3.5. Blood Collection and Polymorphonuclear Leukocytes (PMN) Isolation

Whole blood was obtained with informed consent from healthy volunteers at University “Federico II” in Naples, Italy. Between 08.00 and 09.00 a.m., three healthy fasting donors were subjected to peripheral blood sampling with K3EDTA vacutainers (Becton Dickinson, Plymouth, UK). A discontinuous gradient, consisting of 100% (density 1.1294 g/mL) and 70% (density 1.090 g/mL) isotonic Percoll (Pharmacia, Uppsala, Sweden) in calcium and magnesium-free phosphate buffered saline pH 7.4 (PBS; Sigma-Aldrich, Saint Louis, MO, USA) was used to isolate PMNs [67]. Subsequently, the samples were centrifuged for 20 min at 250× *g* at room temperature. The PMN layer was collected and washed twice in PBS. May-Grunwald Giemsa-stained cytocentrifuge smears were used to determine the isolated PMN purity, while, the trypan blue dye exclusion test was employed to check cell viability. Both ranged between 90% and 95%.

### 3.6. Antioxidant Enzymes Measured in PMN Cells

A commercial kit (BioAssay System, San Diego, CA, USA) was used to determine superoxide dismutase (SOD), catalase (CAT), and glutathione S-transferase (GST) enzymatic activity in PMN cells according to the manufacturer’s recommendations. The activity of enzymes was expressed as U/L [68]. Loroglossol and hircinol were tested at the concentration of 100 µg/mL (3.9–4.1 × 10^−4^ M) and 250 µg/mL (9.8–10 × 10^−4^ M).

### 3.7. Antimicrobial Activity Assays

The presence of antimicrobial molecules in the extracted from Orchid flower was detected using Kirby–Bauer assay [69] against two bacterial strains: *E. coli* DH5α and *S. aureus* ATCC 6538P. Both orchid molecules were used at 50 µg and ampicillin used at 10 µg as a positive control, to inhibit *E. coli* and *S. aureus* cells growth. Another method to evaluate the antimicrobial activity involved the Gram-negative *E. coli* DH5a and Gram-positive *S. aureus* ATCC 6538P strains cell viability counting [70]. Bacterial cells were incubated with loroglossol and hircinol at two different concentrations: 100 µg/mL (3.9–4.1 × 10^−4^ M) and 250 µg/mL (9.8–10 × 10^−4^ M). Bacterial cells immersed in the oil-free buffer represented positive control, while bacterial cells plus 80% DMSO (volume corresponding to the maximum concentration) and buffer represented the negative one [71]. Each experiment was performed in triplicate, and the reported result was an average of three independent experiments (*p* value was <0.05). Subsequently to determine the minimal inhibitory concentration values, assays were performed as previously described elsewhere [72]. Briefly, bacteria were grown to a midlogarithmic phase at 37 °C and then diluted to 1 × 10^6^ CFU/mL in Difco 0.5× Nutrient Broth (Becton-Dickenson, Franklin Lakes, NJ, USA) containing increasing amounts of loroglossol and hircinol. Starting from a compound stock solution, 2-fold serial dilutions were sequentially carried out, accordingly to the broth microdilution method [73]. DMSO was tested at the same concentrations. Following overnight incubation, MIC100 values were determined as the lowest molecule concentrations responsible for no visible bacterial growth.

### 3.8. Cell Cultures and Treatments

Human GC cell lines, AGS (ATCC^®^ CRL-1739™) and KATO-III (ATCC^®^ HTB-103), were purchased from ATCC (Manassas, VA, USA) and routinely cultured in their specific medium according to manufacturer instructions. In particular, AGS were cultured in Dulbecco’s Modified Eagle Medium (DMEM, GIBCO, Grand Island, NY, USA) supplemented with 10% inactivated Fetal Bovine Serum (FBS, GIBCO, Grand Island, NY, USA), 100 U/mL of penicillin and 100 µg/mL of streptomycin. KATO-III cells were grown in Iscove’s Modified Dulbecco’s Medium (IMDM, GIBCO, Grand Island, NY, USA) with 20% inactivated FBS (GIBCO, Grand Island, NY, USA) and 1% penicillin/streptomycin. All cells were incubated at 37 °C in 5% CO_2_ changing medium every 2 days.

Loroglossol and hircinol were first resuspended in DMSO and then diluted in DMEM or IMDM to obtain a stock solution of 10,000 µg/mL. GC cells were incubated with both molecules at two different concentrations: 100 µg/mL (3.9–4.1 × 10^−4^ M) and 250 µg/mL (9.8–10 × 10^−4^ M) for 24 and 48 h. As untreated control, DMSO at final concentration of 0.5% was used.

### 3.9. Cell Proliferation and Apoptosis Analysis

Cell proliferation was assessed using a MTS assay. AGS and KATO-III cells were plated at a density of 5 × 10^3^ cells/well into 96-well plates and grown overnight before treatments with each compound or with an equivalent amount of DMSO for 24 and 48 h. A solution of CellTiter 96^®^ Aqueous MTS Reagent (Promega, Madison, WI, USA) was added to each well measuring, after 1.5 h of incubation at 37 °C, the absorbance at 490 nm. The proliferation rate in each well was calculated using the DMSO treated cells as reference, which represents the 100% of viability. Three replicate wells per point were used to obtain measures of cell proliferation from three independent experiments. Data were reported as mean ± SE. Differences between treatments and control groups were estimated by one sample t test. *p* values < 0.05 were considered significant. Analyses were performed using R software.

To evaluate apoptosis involvement, GC cells were seeded in 6-well plates and cultured to 70% confluence prior to being treated with 100 µg/mL (3.9–4.1 × 10^−4^ M) of both compounds, or with their vehicle, for 48 h. Briefly, cells were harvested, washed twice in cold PBS, centrifuged at 1500 rpm at 4 °C, and then resuspended in Binding Buffer (BD Biosciences, Franklin Lakes, NJ, USA). Cells were double-stained with FITC-Annexin V and propidium iodide (BD Biosciences, Franklin Lakes, NJ, USA) and incubated for 15 min at room temperature in the dark. Number of live, apoptotic, and necrotic cells was detected using a Navios flow cytometer (Beckman Coulter, Miami, FL, USA). Data were analyzed by Kaluza analysis software 2.1. Counts from flow cytometry evaluation of apoptosis were reported as fold increases compared with vehicle controls. Data were reported as mean ± SE. Differences between treatments and control groups were estimated by one sample t test. *p* values < 0.05 were considered significant. Analyses were performed using R software.

## 4. Conclusions

Phytoalexins are produced by plants in response to pathogenic fungal infections. The phytoalexins of orchids are phenanthrene and dihydrophenanthrene, such as hircinol and loroglossol. Phenanthrenes are a promising and expanding group of biologically active natural compounds whose potential has not yet been sufficiently studied [10]. Their antifungal abilities were studied on *Candida lipolytica* as early as 1973. However, very little is known about the antimicrobial activity of these molecules against gram positive and negative bacteria. This research resulted in the isolation and identification of the loroglossol and hircinol from the Orchids *H. robertianum* bulbs and roots. The two molecules showed different bioactivity; they induced an increase in the activity of superoxide dismutase, catalase, and glutathione S-transferase in polymorphonuclear leukocytes; they showed an antimicrobial activity against *E. coli* and *S. aureus*, and they had an anti-proliferative effect on gastric cancer cell lines inducing an apoptosis effect.

Thus, results of this work give new insight into the potential application of these molecules as antimicrobial agents to combat pathogens that are difficult to eradicate and for the discovery of alternatives to antibiotics.

Furthermore, our studies provide the basis for additional investigation of these orchid extracts for the discovery of new potential chemical compounds with anti-cancer properties. Further surveys are needed using in vivo models to determine their effectiveness. For these reasons, these two molecules could be considered promising candidates for pharmaceutical and nutraceutical preparations.

## Figures and Tables

**Table 1:** Comparison of relative viability between the two gastric cancer cell lines.

Time	Dose	Loroglossol			Hircinol		
		AGS	KATO-III	* p *-Value	AGS	KATO-III	* p *-Value
24 h	3.9–4.1 × 10^−4^ M	61.4 ± 2.4	66.9 ± 2.8	0.18	39.4 ± 0.8	56.3 ± 0.9	0.0002
	9.8–10 × 10^−4^ M	37.5 ± 0.4	58.6 ± 1.6	<0.0001	42.9 ± 0.9	48.4 ± 0.2	0.02
48 h	3.9–4.1 × 10^−4^ M	37.2 ± 4.1	53.7 ± 3.6	0.02	31.8 ± 0.2	44.2 ± 1.1	0.006
	9.8–10 × 10^−4^ M	25.8 ± 0.6	32.1 ± 0.9	0.0005	34.3 ± 0.8	44.5 ± 0.3	0.002

Statistical significant assessed by *t*-test.

## Data Availability

The study did not report any data.

## Supplementary Materials

The following are available online at https://www.mdpi.com/article/10.3390/plants10122776/s1, Table S1. Minimum inhibitory concentration values of loroglossol and hircinol against *E. coli* DH5*α* and *S. aureus* ATCC 6538P. Figure S1. Inhibition halo of hircinol and loroglossol against *E. coli* DH5α, *S. aureus* ATCC6538P.

## Sample Availability

Samples of the compounds are available from the authors of the Department STEBICEF, University of Palermo, Italy.
