# Supplementary material for: Dihydrophenanthrenes from a Sicilian Accession of Himantoglossum robertianum (Loisel.) P. Delforge Showed Antioxidant, Antimicrobial, and Antiproliferative Activities
Source: Plants (Basel). 2021 Dec 15;10(12):2776. doi: 10.3390/plants10122776 (PMC8708532; doi:10.3390/plants10122776)
Supplement: Supplementary file 1 [file plants-10-02776-s001.zip › plants-1504955-supplementary.pdf]

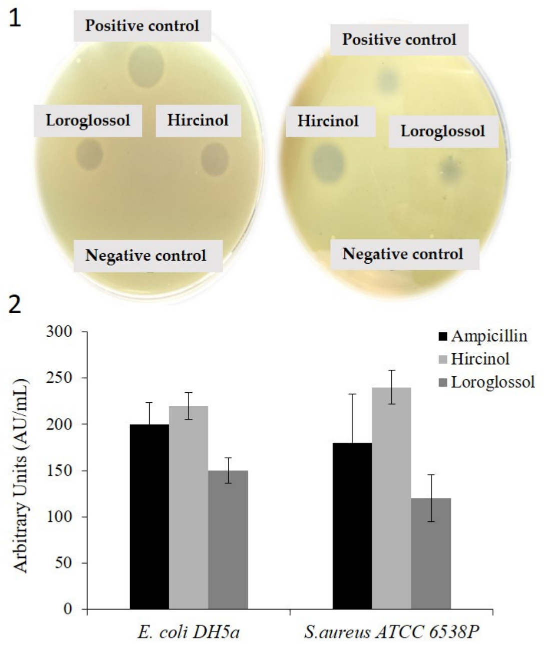

Figure S1. (**1**) Inhibition halo of hircinol and loroglossol against (A) *E. coli DH5α*, (B) *S. aureus ATCC6538P*. Positive control is represented by the antibiotic Ampicillin, negative control is dimethyl sulphoxide (DMSO 80%). (**2**) The inhibition halos showed in panel 1 are expressed in AU/ml (see methods). Values are expressed as average of three different experiments; standard deviations are always less than 10%.

Table S1. Minimum inhibitory concentration values of loroglossol and hircinol against *E. coli* DH5α and *S. aureus* ATCC 6538P

Microrganism	Loroglossol [M]	Hircinol [M]
E. coli DH5α	> 1.2 10^{-3}	1.2 10^{-3}
S. aureus ATCC 6538P	1.2 10^{-3}	4.1 10^{-4}

Values were obtained from a minimum of 3 independent trials.
